# Management of phthalates in Canada and beyond: can we do better to protect human health?

**DOI:** 10.3389/fpubh.2024.1473222

**Published:** 2024-11-13

**Authors:** Matthew J. Renwick, Anette K. Bølling, Erin Shellington, Christopher F. Rider, Miriam L. Diamond, Chris Carlsten

**Affiliations:** ^1^Department of Medicine, University of British Columbia, Vancouver, BC, Canada; ^2^Norwegian Institute of Public Health, Oslo, Norway; ^3^Air Pollution Exposure Laboratory, Division of Respiratory Medicine, Department of Medicine, University of British Columbia, Vancouver, BC, Canada; ^4^Legacy for Airway Health, Vancouver Coastal Health Research Institute, Vancouver, BC, Canada; ^5^Department of Earth Sciences, University of Toronto, Toronto, ON, Canada; ^6^School of the Environment, University of Toronto, Toronto, ON, Canada

**Keywords:** phthalates, chemicals in products, vulnerable populations, child exposure/health, endocrine disruptors, environmental justice, risk management, chemical regulations

## Abstract

Ortho-phthalates (herein referred to as phthalates) are synthetic chemicals used in thousands of different everyday products and materials. Nearly ubiquitous environmental exposure is reflected by phthalate metabolites in the urine of almost all Canadians. However, phthalate exposure tends to be higher amongst people of low socioeconomic status and ethnic minorities. Substantial evidence shows that certain phthalates cause harm to human health, particularly developing fetuses and children. Governments vary in their approach to assessing and managing risks associated with phthalates. Canada continues to take a more permissive stance on phthalate regulations compared to the EU and some US states. We argue that the recent Canadian national risk assessment on phthalates does not appropriately reflect the growing evidence demonstrating harm to human health from phthalate exposure and does not adequately consider the evidence showing higher exposures faced by vulnerable populations. Canadians would benefit from adopting a more stringent regulatory approach to phthalates. Specifically, Canada should expand phthalate restrictions to apply to all consumer products, implement sunset dates toward eliminating the use of existing phthalates, and mandate publicly available evidence of no harm for phthalate alternatives. Canadian alignment on phthalate regulations with the EU and a growing number of US states could encourage other countries to follow suit.

## Introduction

Phthalates are synthetic chemicals used as plasticizers, lubricants, binders, and solvents. Here we use the term “phthalate” to refer to ortho-phthalates. They are used in thousands of products including food packaging, building materials, upholstery, clothing, children's toys, and cosmetic products (1). Phthalates are not chemically bound to their plastic polymers and consequently migrate to the surrounding environment, causing ubiquitous human exposure through inhalation, ingestion, and dermal absorption (2). The Sixth Canadian Health Measures Survey (CHMS) found that almost every Canadian surveyed aged 6–79 between 2018 to 2019 had detectable levels of phthalate metabolites in their urine (3).

In 2020, the Canadian Government published a screening assessment of phthalates to provide an updated cumulative evaluation of their human health and environmental risk. The assessment concluded that all reviewed phthalates, except for one, di-ethyl hexyl phthalate (DEHP), posed no significant human health or ecological risk at present exposure levels. This conclusion is at significant odds with the expanding research documenting phthalate-related human health effects, particularly in fetuses and young children. These serious effects include pre-term birth, abnormalities in the male reproductive system, asthma, infertility, and neurocognitive delay (4).

This discussion illustrates the divergence between Canadian regulations pertaining to phthalates and those of the European Union (EU) and United States (US), and the ensuing implications for harm to human health. First, a summary of current evidence on human exposure to phthalates and health risks is provided. Second, we present an overview of phthalate regulation in Canada and a comparison with the EU and the US. Finally, we discuss the implications of the differing policies taken by Canada, the EU, and the US, as well as provide recommendations for how Canada's regulatory strategy on phthalates could be strengthened to protect human health.

## Phthalate exposure and trends

Phthalates have a wide range of physical and chemical properties that determine their industrial applications and human exposure. They are diesters of phthalic acid and are typically classified into low molecular weight (LMW) and high molecular weight (HMW) phthalates (Table 1). Low molecular weight phthalates have ester side chains of one to four carbons, while HMW phthalates have side chains of five or more (2). For HMW phthalates, direct ingestion occurs as they migrate from plastic packaging into food and beverages, or during mouthing of plastic objects, to which they have been added. Indirect ingestion occurs through unintentional consumption of cosmetic products and, as a result of their migration from products, furnishings and building materials, followed by partitioning into and ingestion of household dust (5, 6). LMW phthalates are more volatile, resulting in exposure mainly through inhalation of indoor air as well as direct exposure from cleaning products and personal care products (7–9). Transdermal uptake occurs mainly from direct skin contact with LMW- and HMW-containing products, such as personal care products and flexible plastics, respectively, but can also occur directly from indoor air (10). Exposure to fetuses can occur as phthalates cross the maternal blood-placenta barrier (11, 12). Phthalates are then rapidly metabolized within ~24 h, before the mono-ester metabolites are excreted primarily through urine, which is the basis for large-scale biomonitoring studies (13).

**Table 1:** Chemical names of ortho-phthalate esters, molecular weights, and CAS number.

**Acronym**	**Chemical name**	**Molecular weight (g mol^−1^)**	**CAS number**
**Low molecular weight phthalates**			
DMP	Dimethyl phthalate	194.2	131-11-3
DEP	Diethyl phthalate	222.2	84-66-2
DiBP	Diisobutyl phthalate	278.3	84-69-5
DBP^a^	Di-n-butyl phthalate	278.3	84-74-2
DMEP	Bis(2-methoxyethyl) phthalate	282.3	117-82-8
**High molecular weight phthalates**			
DPP^b^	Di-n-pentyl phthalate	306.4	131-18-0
DiPP	Disopentyl phthalate	306.4	605-50-5
BBzP	Butyl benzyl phthalate	312.4	85-68-7
DCHP	Dicyclohexyl phthalate	330.4	84-61-7
DHEXP	Dihexyl phthalate	334.4	84-75-3
DOP	Dioctyl Phthalate	390.6	117-81-7
DnOP	Di-n-octyl phthalate	390.6	117-84-0
DEHP	Bis(2-ethylhexyl) phthalate	390.6	117-81-7
DHNUP	Mixture: 1,2-Benzenedicarboxylic acid, di-C7,11-branched and linear alkyl esters	390.6	68515-42-4
DiNP	Diisononyl phthalate	418.6	28553-12-0/68515-48-0
DPHP	Di(2-propylheptyl) phthalate	446.7	53306-54-0
DiDP	Diisodecyl phthalate	446.7	89-16-7
610P	Mixture: 1,2-Benzenedicarboxylic acid, mixed decyl and hexyl and octyl diesters	743	68648-93-1

^a^DBP is synonymous with DnBP.

^b^DPP is synonymous with DPENP.

The global production of phthalates is steadily rising. The international market in 2024 for phthalic anhydride from which phthalates are synthesized, was valued at $4.64 billion USD (14). This market is projected to grow to $5.82 billion USD by 2030, largely attributed to growing consumption of plastic goods. International production volume of di-isononyl phthalate (DiNP) and DEHP, which make up over 75% of the global market share for phthalates, grew by 21% from 2014 to 2019 (15).

Numerous studies have documented a temporal shift towards use of longer-chain phthalates. A US population study by Zota et al. (16) of over 11,000 US adults and children between 2001 and 2010 found that urinary metabolites of di-ethyl phthalate (DEP), di-n-butyl phthalate (DnBP or DBP), butyl benzyl phthalate (BBzP), and DEHP decreased by 20 to 50%, while urinary metabolites of diisobutyl phthalate (DiBP) and DiNP increased by over 100%. Similar trends have been observed in biomonitoring surveys in Canada and Sweden (3, 17). This shift likely reflects changes in HMW phthalate uses prompted by regulatory changes over the last decade that discourage use of “old” phthalates, particularly BBzP and DEHP. In conjunction, “new” phthalates are replacing DEHP, such as didodecyl phthalate (DiDP) and non-ortho phthalate plasticizers, such as 1,2-cyclohexane dicarboxylic acid diisononyl ester (DiNCH). These alternatives have similar product applications to older phthalates and thus similar human exposure routes, but much less is known about the health risks of these alternatives. However, the global rise of DEHP production suggests that populations in low- and middle-income countries may be subjected to increased exposures to DEHP (18).

Biomonitoring data from the US shows that phthalate exposures are tied to ethnicity, with Black Americans generally having higher exposures. As an example, the metabolite of DEP, mono-ethyl phthalate (MEP), was found in higher concentrations in urine from US non-Hispanic Blacks compared to Mexican Americans and non-Hispanic Whites, particularly among children aged 6–11 (19). Follow up studies have reiterated this finding of higher urinary concentrations of phthalate metabolites in African Americans compared to other ethnic cohorts (20, 21). Additionally, African American pregnant women tend to have higher urinary concentrations of phthalate metabolites (22).

Phthalate exposures have also been associated with socioeconomic status. Higher DEHP concentrations were reported among females, urban populations, and low-income US households (23). Moreover, the lowest quartile of socioeconomic class in the US had a higher urinary concentration of mono-benzyl phthalate (MBzP, metabolite of BBzP) but lower concentrations of DEHP metabolites compared to the highest socioeconomic quartile (20). The pattern is similar in Canada. In the Canadian Healthy Infant Longitudinal Development (CHILD) study, phthalate exposure throughout infancy and early life, from 3–4 up to 36 months, was higher among children from lower income than high income families (24). Moreover, children living in newer or larger homes tended to have lower concentrations of phthalate metabolite urinary concentrations, although this finding was not consistent across all study timepoints. Finally, air concentrations of all six measured phthalates were consistently higher in low income social housing relative to higher socio-economic status homes in the Greater Toronto Area (25).

## Human health effects associated with phthalates

There is a large base of literature on the adverse human health effects of ortho-phthalates. Most literature has focused on phthalates with C4 to C10 chain lengths, with particular attention to a few older phthalates, namely DBP, BBzP, and DEHP. Evidence of adverse effects comes from epidemiological studies substantiated by *in vivo* and *in vitro* testing to elucidate plausible mechanisms of action.

Eales et al. (26) summarized the human epidemiologic literature in a recent meta-analysis of 42 reviews, which covered 334 unique studies. They found robust evidence supporting the association between current phthalate exposure and reduced semen quantity, abnormal anogenital distance in boys, neurodevelopmental impairment, and childhood asthma. Modest evidence supported the association between phthalate exposures and low birth weight, endometriosis, decreased testosterone levels, attention deficit hyperactivity disorder, type 2 diabetes, and breast and uterine cancers.

*In vivo* and *in vitro* biologic studies show that phthalates act as potent disrupters of endocrine hormones, which aligns with the strong associations with reproductive, neurodevelopmental, metabolic, and immune disorders noted in epidemiologic studies (27). The primary biologic hypothesis is that phthalate metabolites interfere with ligand signaling systems for multiple different hormone and chemical pathways (18). For instance, DEHP metabolites actively compete with progesterone for binding with progesterone receptors (28).

The strongest biologic evidence for phthalates as toxic endocrine-disrupting compounds is that they directly interfere with testicle function and facilitate testicular dysgenesis (29). *In vitro* rat studies demonstrated that elevated DEHP exposure resulted in apoptosis of Leydig cells, and DBP exposure induced apoptosis of Sertoli cells, both of which are necessary for normal testicular function (30, 31). Prenatal exposure to DBP results in abnormal multinucleated germ cells in male rats, and dicyclohexyl phthalate (DCHP) is linked to malformed seminiferous tubules (32, 33). Other rat studies have identified that prenatal exposure to BBzP, DBP, and DEHP in male rats was associated with a reduction in anogenital distance (34–36).

On an epidemiologic level, studies show that increased phthalate metabolite levels in men are associated with infertility, spermatogenesis impairment, and external genital malformations. For example, a study of 1,247 Chinese men presenting to a reproductive center in Wuhan, China, found that higher phthalate metabolite concentrations in semen were associated with lower semen volume, reduced semen motility, and abnormal spermatic heads and flagella (37). Another key example is Swan's 2015 (38) study that found an elevated concentration of DEHP metabolites in first-trimester pregnant women was inversely associated with anogenital distance in male newborns.

Phthalates potentially disrupt the fine balance between estrogens and androgens required for normal pregnancy. To illustrate, a high urinary concentration of (mono-2-ethylhexyl phthalate) MEHP, a DEHP urinary metabolite, was associated with 2.9 times higher risk of pregnancy loss, compared to the lowest urinary concentrations (39). Phthalate exposure was also associated with significantly increased odds of preterm birth, spontaneous preterm birth, and a high incidence of pregnancy-induced hypertension (40, 41).

There is mechanistic evidence to support the hypothesis that phthalate exposure also disrupts the hypothalamic-pituitary-gonadal, adrenal and thyroid axes, as well as directly interfering with neural receptors (42). This may explain the weak association between prenatal or childhood phthalate exposure and neurodevelopmental impairments, such as autism and attention deficit hyperactivity disorder (43–45). Furthermore, metabolic effects such as diabetes may be mediated by errant activation of metabolic transcription factors, increased oxidative and inflammatory stress, antiandrogen effects, and epigenetic impacts. A systematic review and meta-analysis regarding cardiometabolic risks associated with child and adolescent exposures to phthalates concluded that LMW and HMW phthalate metabolites in children and adolescents were positively associated with increased body-mass index and hypertension, but not waist circumference, serum high density lipoprotein, and serum triglyceride levels (46).

Finally, phthalates and their metabolites have been shown to affect the immune system by dysregulating gene expression, interfering with immune enzyme activity, and disrupting immune signaling pathways (47). This may explain why phthalate exposure is associated with an increased propensity for the development of allergic diseases. Controlled exposure to DBP exacerbated allergen-induced lung function decline by altering lung immunology in a recent randomized crossover study in humans (48). In Canadian children, DEHP exposure at age three to four months was associated with an increased risk of developing asthma and recurrent wheeze by the age of five (49). Overall, the entire body of research projects a weight-of-evidence toward there being significant human adverse effects from phthalates at current exposure levels.

## Canadian regulation of phthalates

To understand the phthalate regulations in Canada, it is helpful to review the historical context through which chemicals have come to be nationally managed. Health Canada (HC), and Environment and Climate Change Canada (ECCC) are the federal departments responsible for risk assessment and management of chemical substances (Supplementary Table 1) (50). The Canadian Environment Protection Act (CEPA) of 1999 is the federal legislation that mandates HC and ECCC to evaluate new and existing chemicals registered on Canada's Domestic Substance List (50). Health Canada and ECCC maintain a Priority Substance List (PSL) of chemicals that are evaluated as to whether they meet criteria as a Schedule 1 toxic chemical, which would require a dedicated risk management strategy (51).

Assessments of the first PSL were completed by 1994 with four phthalates included: DEHP, DBP, BBzP, and di-n-octyl phthalate (DnOP) (52). DEHP was the only phthalate that fulfilled the criteria for listing on Schedule 1 toxic chemical to humans (53). The risk management measures for DEHP were to: (i) add it to the Cosmetics Ingredients Hotlist to prevent its presence in any concentration in cosmetic products, (ii) implement regulations regarding use in medical devices, and (iii) limit its use to 0.1% by weight in vinyl children's toys and childcare articles. Assessments of the second PSL were completed by 2000 and no additional phthalates were defined as toxic (54).

The Chemicals Management Plan (CMP) was formed in 2006 to facilitate further review of earmarked chemicals and to apply statutory and non-regulatory tools to mitigate risks (55). The CMP implemented the Substance Groupings Initiative as a way of handling some of the 4,000 chemicals and streamlining the assessment process, informing substitution recommendations, and improving stakeholder engagement. There were nine groupings with one being the Phthalate Substance Grouping (PSG), where the goal was to assess cumulative risk from combined exposures. The PSG included 14 phthalates earmarked for individual assessments (56).

In 2015, four “State of the Science” reports were published for the PSG (57–60). In recognition of the potential combined risk of numerous phthalate exposures, a “Proposed Approach for Cumulative Risk Assessment of Phthalates” was published (61). These documents along with multi-stakeholder input formed the groundwork for the Final Screening Assessment of the PSG in 2020 (56). It examined the 14 phthalates of the PSG, as well as 14 additional phthalates on the Domestic Substance List, as part of a broader cumulative risk assessment for the entire chemical class. Four of these phthalates (DBP, BBzP, DnOP, and DEHP) had been previously assessed as part of the first or second PSL.

The 2020 Screening Assessment concluded that, individually, the 14 phthalates “are not harmful to human health or to the environment at levels of exposure considered in the assessment” (62). In other words, they did not meet the criteria of being “Schedule 1 toxic” because they posed a low risk of harm to Canadians and the environment (56). DEHP remained the only phthalate deemed to be “Schedule 1 toxic,” with the revised assessment concluding that it could also pose environmental harm. As part of a CMP risk management program, DEHP is subject to biannual Performance Measurement Evaluations that document DEHP biomonitoring data from the Canada Health Measure Survey, due to DEHP's earlier designation of posing a threat to human health (63). The assessment's cumulative risk analysis determined that there was no ecological or human health concern from the cumulative exposure to the 28 phthalates, largely due to the relatively low exposures to these chemicals in the populations that were assessed (56).

The Canada Consumer Product Safety Act (CCPSA) serves as an important statute through which restrictions are imposed on phthalates deemed to pose a risk to human health. Its Phthalate Regulations section limits the use of six phthalates (DBP, BBzP, DEHP, DnOP, DiNP, and DiDP) in listed children's products. DBP, BBzP, and DEHP are limited to a maximum concentration of 1,000 mg/kg (0.1% by weight) in the soft vinyl of toys and articles, including those “intended to facilitate the relaxation, sleep, hygiene, feeding, sucking or teething of a child under the age of four,” while DnOP, DiNP, DiDP are limited to that concentration in any toy or child care products that could be mouthed (64).

## International comparison of phthalate regulations

In the EU, the Registration, Evaluation, Authorization, and Restriction of Chemicals (REACH) is the primary legislation protecting the public and the environment from exposures to harmful chemicals (Supplementary Table 1) (50). The European Chemicals Agency (ECHA) is the government body managing the use of these toxic chemicals. As in Canada, ECHA maintains a list of high-priority chemicals, called the Candidate List of Substances of Very High Concern, that are marked for more rigorous screening and management plans for products in the EU market (65).

The REACH Authorization List (Annex XIV) is a narrowed list of Substances of Very High Concern that have a sunset date, after which their continued use in the EU is prohibited (66). The purpose is to push European manufacturers to replace the use of toxic substances with safer alternatives. Companies seeking to use a listed chemical for production in the EU after its sunset date must comply with specified restrictions and demonstrate that an appropriate risk management plan is in place, no suitable alternative exists, and that the benefit of its continued use outweighs the risks. The list currently includes 14 phthalates, most of whose sunset dates have passed (66). There have been no recent requests for authorization for listed phthalates, suggesting that safer alternatives are readily available and that this is an effective means to phase out their use (67). But REACH Authorization does not apply to imported articles, leaving a regulatory and safety gap (68).

This gap is partly covered by Annex VII of REACH, which sets specific restrictions on phthalate use in consumer products (Table 2) manufactured domestically or imported into the EU. Use of DBP, DiBP, BBzP, and DEHP must be limited to under 0.1% by weight in all consumer products, not just childcare articles (Table 2) (69). However, numerous uses are exempt from these restrictions, including products for industrial or agricultural use, motor vehicles, aircrafts, certain medical devices, electrical and electronic equipment, and food contact materials, which are regulated separately. Additionally, three long-chain phthalates, DnOP, DiNP and DiDP, are now restricted to 0.1% by weight in toys and articles that could be placed in a child's mouth (87). The EU has also banned certain phthalates bis(2-methoxyethyl) phthalate (DMEP), DBP, Di-n-pentyl phthalate (DPP), Diisopentyl phthalate (DIPP), BBzP, and DEHP in cosmetic products under the Cosmetics Products Regulation (70).

**Table 2:** Simplified comparison of phthalate restrictions for children's toys, cosmetics, and food packaging between Canada, the EU, and US.

	**Children's toys**	**Cosmetics**	**Food packing**	**Additional regulations**
Canada	BBzP, DBP, DEHP, DiNP, DiDP, DnOP limited to < 0.1% of weight in child toys	DEHP banned from use in cosmetics	None	BBzP, DBP, DEHP limited to < 0.1% of weight in children's products intended to promote relaxation and sleep (e.g., mattresses)
EU	BBzP, DBP, DEHP, DiBP, DiNP, DiDP, DnOP limited to < 0.1% of weight in child toys	BBzP, DBP, DEHP, DiBP are banned from use in cosmetics	Migration limits for the following food contact materials: DEHP < 1.5 mg/kg DBP < 0.3 mg/kg BBzP < 30 mg/kg DiDP and DiNP combined limit of 9 mg/kg	BBzP, DBP, DEHP, DiBP limited to < 0.1% of weight in most consumer products
US	BBzP, DBP, DEHP, DiBP, DiNP, DPP, DHEXP, DCHP limited to < 0.1% of weight in child toys	No national restrictions	No national restrictions; some state restrictions apply	DEHP limited to < 0.006 mg/L in drinking water

Separate EU regulations limit phthalate use in food packaging or materials in high contact with non-fatty foods (71). These restrictions reflect migration limits, which are the maximum permitted quantity of a substance that can migrate from a food package or container into the food. The specific migration limits are: DBP < 0.3 mg/kg, BBzP < 30 mg/kg, DEHP < 1.5 mg/kg, and DiNP and DiDP combined limit of 9 mg/kg.

In the US, the United States Environmental Protection Agency (EPA) is responsible for evaluating and monitoring the safety of chemicals (Supplementary Table 1) (50). The Toxic Substances Control Act (TSCA) Work Plan was developed by the US EPA in 2014 to monitor chemicals identified as higher risk based on toxicity, exposure, persistence, or bioaccumulation. Ninety substances are part of this Work Plan, of which seven are phthalates (DBP, DiBP, BBzP, DEHP, DnOP, DiNP, DiDP). These chemicals are then further stratified, with the highest priority substances assigned for further risk evaluation. Only 10 substances, none of which are phthalates, have been fully assessed (50). The seven phthalates are currently in the early stages of TSCA risk evaluation (72). Separately, the EPA is entrusted with ensuring DEHP meets the maximum water concentration of 0.006 mg/L through the Safe Drinking Water Act (73).

The US Food and Drug Administration (FDA) monitors the safety of cosmetic products and takes the stance that insufficient evidence exists to draw clear connections between phthalates in cosmetics and human health effects (74). Consequently, phthalates are not banned from use in cosmetic products marketed in the US. If used in a cosmetic product, phthalates must be listed as an ingredient on the packaging (50). However, this does not apply to fragranced personal care products, which often contain LMW phthalates under the label “parfum.” The same situation pertains to Canada, except for the ban of DEHP in cosmetics. The FDA also regulates phthalates used as food additives and in food contact materials (75). In 2022, the FDA rescinded authorization of 23 obsolete phthalates previously allowed as food additives, but upheld the decision to permit continued use of eight key phthalates (DBP, DiBP, BBzP, DCHP, di-n-hexyl phthalate (DHEXP), DEHP, DiOP, DiNP) in food contact materials (76).

Finally, the US Consumer Product Safety Commission (CPSC) limits the weighted quantity of eight phthalates (DBP, DiBP, DPP, BBzP, DCHP, DHEXP, DEHP, DiNP) to < 0.1% by weight in all children's toys and articles (Table 2) (50). This threshold is identical to that used in Canada and the EU, but the selection of phthalates and the products differ, containing several alternative, HMW phthalates.

## Discussion

The primary basis for Canada's current regulatory strategy on phthalates is the 2020 Screening Risk Assessment, which concluded that the risk posed by phthalates is not of significant concern to Canadians or the environment at current levels. The caveat to this is DEHP which was confirmed to be “CEPA” toxic to human health and the environment, and thus subject to risk management measures. Consequently, DEHP exposure is monitored through the Canadian Health Measures Survey by measuring urinary concentrations of phthalate metabolites, and targeted food monitoring (62). The only formal restriction on phthalates is through the CCPSA which limits certain phthalates to a maximum concentration in children's toys and articles. In addition, DEHP is prohibited from use in cosmetics.

In contrast, the EU employs more stringent regulations, implying that their underlying risk assessments identified phthalate exposure and toxic effects to be of greater health concern. The REACH Authorization list applies a sunset date on high-risk phthalates, forcing domestic companies to seek alternatives to harmful phthalates. But REACH Authorization does not extend to imported goods and allows for numerous exemptions, which leaves a gap in regulation (68). Additional REACH restrictions limit phthalate concentrations in children's toys and articles, like in Canada, but they also extend more broadly to many consumer products. These restrictions even apply to imported goods. Temporal trends in phthalate biomonitoring and indoor levels in the EU and North America indicate that such regulations are an effective strategy for reducing the use of phthalates, such as BBzP.

The US, with its multi-agency legislative complexities, is slow to develop a cohesive phthalate strategy and has stalled on TSCA's risk assessment phase of high-priority phthalates. The FDA, which holds significant authority to limit the use of phthalates in cosmetics and food packaging, has taken the stance that the lack of firm causality between phthalate exposure and human harm precludes stricter regulatory action. However, public and political concern continue to grow as illustrated by recurrent petitions and lawsuits filed against the FDA and formal requests from a US House Representative (77). In response to the lack of regulation at the federal level, individual states have implemented their own restrictions. For example, Maine has banned the use of phthalates in food packaging, while Minnesota, Michigan, New Jersey, and New York are now considering the regulation of phthalates (78, 79). As with other chemicals (e.g., per- and polyfluorinated alkyl substances or PFAS), legislation passed by certain states confronts manufacturers with a patchwork of restrictions.

Canada's regulations pertaining to DEHP appear to be effective, as seen by decreasing exposures among those Canadians being monitored. However, reductions in DEHP are accompanied by increased use and exposure to longer chain phthalates, such as DnOP, DiNP and DiDP, representing a switch from chemicals with well-known to poorly known toxicity (80). Although Canada's CMP has provisions to re-evaluate updated evidence regarding exposures and toxicity, the reality is that its abilities to conduct these re-assessments are limited. Further, concerns that the conclusions drawn from the 2020 Screening Risk Assessment may not have adequately captured actual population risk comes from insufficient risk characterization of vulnerable populations, which is a consideration now enshrined in the Bill S-5 that updated CEPA (81). Here, a vulnerable population includes people with greater biological susceptibility, such as the fetus, and greater potential for exposure, such as those with lower socio-economic status. These considerations tie in with the evidence of fetal exposure leading to greater susceptibility to endocrine modulating effects and higher exposures of populations with lower socio-economic status. Although the limitations of the 2020 assessment were in part due to a lack of Canadian data on vulnerability to higher exposures, recent publications suggest that Canadians of low socio-economic status are subjected to higher exposures (24, 25). In contrast, no Canadian data exist for exposures in other vulnerable populations, like Indigenous and racialized populations. Canadian and US data suggest that the inequity in exposure may be linked to usage patterns of cheaper building materials and indoor products, like mattresses, as well as increased consumption of packaged, processed food (25, 49, 82).

As recent Canadian governmental reports concluded that dietary exposure to DEHP and other phthalates poses no concern for human health, there are no Canadian regulations on phthalates in food contact materials. However, none of the recent Canadian monitoring programs target vulnerable populations for whom food intake could vary from the “average” Canadian diet (83). The lack of regard for potential risks arising from diet is at odds with the large body of data showing that packaged and processed foods lead to higher exposures to HMW phthalates, especially for some vulnerable populations (5, 16).

From a broader perspective, low- and middle-income countries are often not able to enact formal safety regulations on chemicals. These smaller markets tend to adopt standards set by larger markets, primarily out of prioritizing trade as opposed to an underlying health or environmental basis (84). Canadian alignment on phthalate regulations with the EU and some US states could further encourage low- and middle-income markets to follow suit. Global agreement on chemical safety is a key component of the United Nation's Strategic Approach to International Chemicals Management (85) and its successor, the Global Framework on Chemicals (86).

## Conclusion and recommendations

Canada's 2020 Screening Assessment was a step toward building a national understanding of phthalates and their risk to Canadians. However, we submit that new information on phthalate exposures, specifically those experienced by vulnerable populations, together with the Government of Canada's new commitment to consider vulnerable populations in its chemical management approach, merits the re-assessment of phthalate risk and risk management measures. Our analysis highlights that updated Canadian regulations should draw from the more protective measures implemented by the EU and now some US states.

We have three practical recommendations for improving the management of phthalates in Canada, drawing from the EU's list of specific phthalates:

1. Extend specific phthalate restrictions to all consumer products (including cosmetics and personal care products), furnishings, building materials, food contact products and products used by children,
2. Implement an authorization list with sunset dates toward elimination of phthalates manufactured within or imported into Canada, and
3. Require clear, publicly available evidence of no harm for phthalate alternatives.

These recommendations align Canada with global ambitions to reduce plastic additives, set a positive precedent for countries grappling with phthalate regulation, and better protect human health, especially among vulnerable populations.

## Data Availability

The original contributions presented in the study are included in the article/Supplementary material, further inquiries can be directed to the corresponding authors.

## References

[1] US Environmental Protection Agency. Biomonitoring: Phthalates. (2014). Available at: https://www.epa.gov/americaschildrenenvironment/biomonitoring-phthalates-report-contents (accessed November 24, 2021).

[2] National Research Council. Phthalates and Cumulative Risk Assessment: The Task Ahead. Phthalates and Cumulative Risk Assessment. Washington, DC: National Academies Press (2008). 25009926

[3] Health Canada. Sixth Report on Human Biomonitoring of Environmental Chemicals in Canada: Results of the Canadian Health Measures Survey Cycle 6 (2018 - 2019). Ottawa (2021).

[4] Wang Y Qian H. Phthalates and their impacts on human health. Healthcare. (2021) 9. 10.3390/healthcare9050603 34069956 PMC8157593

[5] Rudel RA Gray JM Engel CL Rawsthorne TW Dodson RE Ackerman JM. Food packaging and bisphenol A and bis(2-ethyhexyl) phthalate exposure: findings from a dietary intervention. Environ Health Perspect. (2011) 119:914–20. 10.1289/ehp.1003170 21450549 PMC3223004

[6] Yang C Harris SA Jantunen LM Kvasnicka J Nguyen L V Diamond ML. Phthalates: relationships between air, dust, electronic devices, and hands with implications for exposure. Environ Sci Technol. (2020) 54:8186–97. 10.1021/acs.est.0c00229 32539399

[7] Jaakkola JJK Knight TL. The role of exposure to phthalates from polyvinyl chloride products in the development of asthma and allergies: a systematic review and meta-analysis. Environ Health Perspect. (2008) 116:845–53. 10.1289/ehp.10846 18629304 PMC2453150

[8] Rudel RA Camann DE Spengler JD Korn LR Brody JG. Phthalates, alkylphenols, pesticides, polybrominated diphenyl ethers, and other endocrine-disrupting compounds in indoor air and dust. Environ Sci Technol. (2003) 37:4543–53. 10.1021/es0264596 14594359

[9] Berger KP Kogut KR Bradman A She J Gavin Q Zahedi R. Personal care product use as a predictor of urinary concentrations of certain phthalates, parabens, and phenols in the HERMOSA study. J Expo Sci Environ Epidemiol. (2018) 29:21–32. 10.1038/s41370-017-0003-z 29317738 PMC6037613

[10] Weschler CJ Bekö G Koch HM Salthammer T Schripp T Toftum J. Transdermal uptake of diethyl phthalate and di(n-butyl) phthalate directly from air: experimental verification. Environ Health Perspect. (2015) 123:928. 10.1289/ehp.1409151 25850107 PMC4590762

[11] Main KM Mortensen GK Kaleva MM Boisen KA Damgaard IN Chellakooty M. Human breast milk contamination with phthalates and alterations of endogenous reproductive hormones in infants three months of age. Environ Health Perspect. (2006) 114:270. 10.1289/ehp.8075 16451866 PMC1367843

[12] Qian Y Shao H Ying X Huang W Hua Y. The endocrine disruption of prenatal phthalate exposure in mother and offspring. Front Public Health. (2020) 8:366. 10.3389/fpubh.2020.00366 32984231 PMC7483495

[13] Wang Y Zhu H Kannan K. A review of biomonitoring of phthalate exposures. Toxics. (2019) 7. 10.3390/toxics7020021 30959800 PMC6630674

[14] Grand View Research. Market Analysis Report: Phthalic Anhydride. (2024). Available at: https://www.grandviewresearch.com/industry-analysis/phthalic-anhydride-market# (accessed October 2, 2024).

[15] Steady growth predicted in global markets for DINP and DOP phthalate plasticizers. Addit Polym. (2015) 2015:11. 10.1016/S0306-3747(15)30127-5

[16] Zota AR Calafat AM Woodruff TJ. Temporal trends in phthalate exposures: findings from the National Health and Nutrition Examination Survey, 2001-2010. Environ Health Perspect. (2014) 122:235–41. 10.1289/ehp.1306681 24425099 PMC3948032

[17] Shu H Jönsson BA Gennings C Svensson Å Nånberg E Lindh CH. Temporal trends of phthalate exposures during 2007–2010 in Swedish pregnant women. J Expo Sci Environ Epidemiol. (2018) 28:437–47. 10.1038/s41370-018-0020-6 29472621

[18] Benjamin S Masai E Kamimura N Takahashi K Anderson RC Faisal PA. Phthalates impact human health: epidemiological evidences and plausible mechanism of action. J Hazard Mater. (2017) 340:360–83. 10.1016/j.jhazmat.2017.06.036 28800814

[19] Silva MJ Barr DB Reidy JA Malek NA Hodge CC Caudill SP. Urinary levels of seven phthalate metabolites in the U.S. population from the National Health and Nutrition Examination Survey (NHANES) 1999-2000. Environ Health Perspect. (2004) 112:331. 10.1289/ehp.6723 14998749 PMC1241863

[20] Kobrosly RW Parlett LE Stahlhut RW Barrett ES Swan SH. Socioeconomic factors and phthalate metabolite concentrations among United States women of reproductive age. Environ Res. (2012) 115:11–7. 10.1016/j.envres.2012.03.008 22472009

[21] Huang T Saxena AR Isganaitis E James-Todd T. Gender and racial/ethnic differences in the associations of urinary phthalate metabolites with markers of diabetes risk: National Health and Nutrition Examination Survey 2001-2008. Environ Health. (2014) 13. 10.1186/1476-069X-13-6 24499162 PMC3922428

[22] Wenzel AG Brock JW Cruze L Newman RB Unal ER Wolf BJ. Prevalence and predictors of phthalate exposure in pregnant women inCharleston, SC. Chemosphere. (2018) 193:394. 10.1016/j.chemosphere.2017.11.019 29154114 PMC6282186

[23] Koo JW Parham F Kohn MC Masten SA Brock JW Needham LL. The association between biomarker-based exposure estimates for phthalates and demographic factors in a human reference population. Environ Health Perspect. (2002) 110:405–10. 10.1289/ehp.02110405 11940459 PMC1240804

[24] Navaranjan G Takaro TK Wheeler AJ Diamond ML Shu H Azad MB. Early life exposure to phthalates in the Canadian Healthy Infant Longitudinal Development (CHILD) study: a multi-city birth cohort. J Expo Sci Environ Epidemiol. (2019) 30:70–85. 10.1038/s41370-019-0182-x 31641275

[25] Wan Y Diamond ML Siegel JA. Elevated concentrations of semivolatile organic compounds in social housing multiunit residential building apartments. Environ Sci Technol Lett. (2020) 7:191–7. 10.1021/acs.estlett.0c00068

[26] Eales J Bethel A Galloway T Hopkinson P Morrissey K Short RE. Human health impacts of exposure to phthalate plasticizers: an overview of reviews. Environ Int. (2022) 158:106903. 10.1016/j.envint.2021.106903 34601394

[27] Diamanti-Kandarakis E Bourguignon JP Giudice LC Hauser R Prins GS Soto AM. Endocrine-disrupting chemicals: an endocrine society scientific statement. Endocr Rev. (2009) 30:293. 10.1210/er.2009-0002 19502515 PMC2726844

[28] Sheikh IA. Stereoselectivity and the potential endocrine disrupting activity of di-(2-ethylhexyl)phthalate (DEHP) against human progesterone receptor: a computational perspective. J Appl Toxicol. (2016) 36:741–7. 10.1002/jat.3302 26879776

[29] Hlisníková H Petrovičová I Kolena B Šidlovská M Sirotkin A. Effects and mechanisms of phthalates' action on reproductive processes and reproductive health: a literature review. Int J Environ Res Public Health. (2020) 17:1–37. 10.3390/ijerph17186811 32961939 PMC7559247

[30] Sun Y Shen J Zeng L Yang D Shao S Wang J. Role of autophagy in di-2-ethylhexyl phthalate (DEHP)-induced apoptosis in mouse Leydig cells. Environ Pollut. (2018) 243:563–72. 10.1016/j.envpol.2018.08.089 30216888

[31] Wang H Wang J Zhang J Jin S Li H. Role of PI3K/AKT/mTOR signaling pathway in DBP-induced apoptosis of testicular sertoli cells *in vitro*. Environ Toxicol Pharmacol. (2017) 53:145–50. 10.1016/j.etap.2017.05.013 28578144

[32] Li L Bu T Su H Chen Z Liang Y Zhang G. *In utero* exposure to diisononyl phthalate caused testicular dysgenesis of rat fetal testis. Toxicol Lett. (2015) 232:466–74. 10.1016/j.toxlet.2014.11.024 25445723

[33] Mahood IK McKinnell C Walker M Hallmark N Scott H Fisher JS. Cellular origins of testicular dysgenesis in rats exposed in utero to di(n-butyl) phthalate. Int J Androl. (2006) 29:148–54. 10.1111/j.1365-2605.2005.00574.x 16466534

[34] Tyl RW Myers CB Marr MC Fail PA Seely JC Brine DR. Reproductive toxicity evaluation of dietary butyl benzyl phthalate (BBP) in rats. Reprod Toxicol. (2004) 18:241–64. 10.1016/j.reprotox.2003.10.006 15019722

[35] Parks LG Ostby JS Lambright CR Abbott BD Klinefelter GR Barlow NJ. The plasticizer diethylhexyl phthalate induces malformations by decreasing fetal testosterone synthesis during sexual differentiation in the male rat. Toxicol Sci. (2000) 58:339–49. 10.1093/toxsci/58.2.339 11099646

[36] Foster PMD Cattley RC Mylchreest E. Effects of Di-n-butyl phthalate (DBP) on male reproductive development in the rat: implications for human risk assessment. Food Chem Toxicol. (2000) 38(1 Suppl):S97–99. 10.1016/S0278-6915(99)00128-3 10717378

[37] Wang YX Zeng Q Sun Y Yang P Wang P Li J. Semen phthalate metabolites, semen quality parameters and serum reproductive hormones: a cross-sectional study in China. Environ Pollut. (2016) 211:173–82. 10.1016/j.envpol.2015.12.052 26766535

[38] Swan SH. Team the TS, Sathyanarayana S, Team the TS, Barrett ES, Team the TS, et al. First trimester phthalate exposure and anogenital distance in newborns. Hum Reprod. (2015) 30:963–72. 10.1093/humrep/deu363 25697839 PMC4359397

[39] Toft G Jönsson BAG Lindh CH Jensen TK Hjollund NH Vested A. Association between pregnancy loss and urinary phthalate levels around the time of conception. Environ Health Perspect. (2012) 120:458–63. 10.1289/ehp.1103552 22113848 PMC3295336

[40] Ferguson KK McElrath TF Meeker JD. Environmental phthalate exposure and preterm birth. JAMA Pediatr. (2014) 168:61–7. 10.1001/jamapediatrics.2013.3699 24247736 PMC4005250

[41] Bedell SM Lyden GR Sathyanarayana S Barrett ES Ferguson KK Santilli A. First- and third-trimester urinary phthalate metabolites in the development of hypertensive diseases of pregnancy. Int J Environ Res Public Health. (2021) 18. 10.3390/ijerph182010627 34682373 PMC8536149

[42] Hlisníková H Petrovičová I Kolena B Šidlovská M Sirotkin A. Effects and mechanisms of phthalates' action on neurological processes and neural health: a literature review. Pharmacol Rep. (2021) 73:386–404. 10.1007/s43440-021-00215-5 33460007

[43] Jeddi MZ Janani L Memari AH Akhondzadeh S Yunesian M. The role of phthalate esters in autism development: a systematic review. Environ Res. (2016) 151:493–504. 10.1016/j.envres.2016.08.021 27567353

[44] Nilsen FM Tulve NS. A systematic review and meta-analysis examining the interrelationships between chemical and non-chemical stressors and inherent characteristics in children with ADHD. Environ Res. (2020) 180:108884. 10.1016/j.envres.2019.108884 31706600 PMC6937727

[45] Choi G Villanger GD Drover SSM Sakhi AK Thomsen C Nethery RC. Prenatal phthalate exposures and executive function in preschool children. Environ Int. (2021) 149:106403. 10.1016/j.envint.2021.106403 33524667 PMC7945722

[46] Golestanzadeh M Riahi R Kelishadi R. Association of exposure to phthalates with cardiometabolic risk factors in children and adolescents: a systematic review and meta-analysis. Environ Sci Pollut Res Int. (2019) 26:35670–86. 10.1007/s11356-019-06589-7 31728953

[47] Zhang Y Lyu L Tao Y Ju H Chen J. Health risks of phthalates: a review of immunotoxicity. Environ Pollut. (2022) 313:120173. 10.1016/j.envpol.2022.120173 36113640

[48] Maestre-Batlle D Huff RD Schwartz C Alexis NE Tebbutt SJ Turvey S. Dibutyl phthalate augments allergen-induced lung function decline and alters human airway immunology. A randomized crossover study. Am J Respir Crit Care Med. (2020) 202:672–80. 10.1164/rccm.201911-2153OC 32320637

[49] Navaranjan G Diamond ML Harris SA Jantunen LM Bernstein S Scott JA. Early life exposure to phthalates and the development of childhood asthma among Canadian children. Environ Res. (2021) 197:110981. 10.1016/j.envres.2021.110981 33691158

[50] Eichler CMA Cohen Hubal EA Little JC. Assessing human exposure to chemicals in materials, products and articles: the international risk management landscape for phthalates. Environ Sci Technol. (2019) 53:13583–97. 10.1021/acs.est.9b03794 31617344 PMC9311451

[51] Canadian Environmental Protection Act 1999 - Canada.ca. Available at: https://www.canada.ca/en/environment-climate-change/services/canadian-environmental-protection-act-registry/publications/canadian-environmental-protection-act-1999.html (accessed November 24, 2021).

[52] Health Canada Environment and Climate Change Canada. First Priority Substances List (PSL1). (2013). Available at: https://www.ec.gc.ca/ese-ees/default.asp?lang=En&n=95d719c5-1 (accessed February 21, 2022).

[53] Environment Canada and Health Canada. Priority Substances List Assessment Report: Bis(2-ethylhexyl) Phthalate. (1994).

[54] Health Canada Environment and Climate Change Canada. Second Priority Substances List (PSL2) Assessments. Ottawa (2009). Available at: https://www.canada.ca/en/health-canada/services/environmental-workplace-health/reports-publications/environmental-contaminants/second-priority-substances-list-psl2-assessments-priority-substances-assessment-program-environmental-contaminants.html (accessed February 21, 2022).

[55] Government of Canada. Chemicals Management Plan. (2016). Available at: https://www.canada.ca/en/health-canada/services/chemical-substances/chemicals-management-plan.html (accessed February 21, 2022).

[56] Health Canada. Screening assessment - Phthalate substance grouping. (2020). Available at: https://www.canada.ca/en/environment-climate-change/services/evaluating-existing-substances/screening-assessment-phthalate-substance-grouping.html (accessed November 24, 2021).

[57] Environment Canada and Health Canada. State of the Science Report Phthalate Substance Grouping: Short-chain Phthalate Esters. Ottawa (2015).

[58] Environment Canada and Health Canada. State of the Science Report Phthalate Substance Grouping: Medium-Chain Phthalate Esters Chemical Abstracts Service Registry Numbers. (Ottawa) (2015).

[59] Environment Canada and Health Canada. State of the Science Report Phthalates Substance Grouping: Long-chain Phthalate Esters. Ottawa (2015).

[60] Environment Canada and Health Canada. State of the Science Report Phthalate Substance Grouping: Diisononyl Phthalate (DINP). Ottawa (2015).

[61] Environment Canada and Health Canada. Proposed Approach for Cumulative Risk Assessment of Certain Phthalates under the Chemicals Management Plan. Ottawa (2015).

[62] Government of Canada. Phthalate Substance Grouping – Information Sheet. (2021) Available at: https://www.canada.ca/en/health-canada/services/chemical-substances/fact-sheets/chemicals-glance/phthalates.html (accessed July 14, 2022).

[63] Health Canada. Performance measurement evaluation for risk management of bis(2-ethylhexyl) phthalate (DEHP), health component. Available at: https://www.canada.ca/en/environment-climate-change/services/evaluating-existing-substances/evaluation-risk-management-dehp.html (accessed November 27, 2021).

[64] Canada Consumer Product Safety Act. Phthalates Regulations. (2016). Available at: https://laws-lois.justice.gc.ca/eng/regulations/SOR-2016-188/page-1.html (accessed November 24, 2021).

[65] European Chemicals Agency. Candidate List of substances of very high concern for Authorisation - ECHA. Available at: https://echa.europa.eu/candidate-list-table?p_p_id=disslists_WAR_disslistsportlet&p_p_lifecycle=0&p_p_state=normal&p_p_mode=view&_disslists_WAR_disslistsportlet_haz_detailed_concern=&_disslists_WAR_disslistsportlet_orderByCol=dte_inclusion&_disslists_WAR_disslistsportlet_substance_identifier_field_key=&_disslists_WAR_disslistsportlet_orderByType=desc&_disslists_WAR_disslistsportlet_dte_inclusionFrom=&_disslists_WAR_disslistsportlet_dte_inclusionTo=&_disslists_WAR_disslistsportlet_doSearch=&_disslists_WAR_disslistsportlet_deltaParamValue=50&_disslists_WAR_disslistsportlet_resetCur=false&_disslists_WAR_disslistsportlet_delta=200 (accessed April 16, 2022).

[66] European Chemicals Agency. Phthalates. Available at: https://echa.europa.eu/hot-topics/phthalates (accessed April 16, 2022).

[67] European Chemicals Agency Danish Environmental Protection Agency. Annex XV Restriction Report: Proposal for a restriction on four phthalates (DEHP, BBP, DBP, DIBP). (2016). Available at: https://echa.europa.eu/documents/10162/2700f4f2-579a-1fbe-2c23-311706a3e958 (accessed June 25, 2024).

[68] Schenten J Führ M. SVHC in imported articles: reach authorisation requirement justified under WTO rules. Environ Sci Eur. (2016) 28:21. 10.1186/s12302-016-0090-9 27752454 PMC5044971

[69] European Commission. Commission Regulation (EU) 2018/2005. (2018). Available at: https://eur-lex.europa.eu/legal-content/EN/TXT/?uri=uriserv:OJ.L_.2018.322.01.0014.01.ENG&toc=OJ:L:2018:322:TOC (accessed November 27, 2021).

[70] European Commission. Regulation (EC) No 1223/2009 of the European Parliament and of the Council of 30 November 2009 on cosmetic products. (2009). Available at: https://eur-lex.europa.eu/eli/reg/2009/1223/oj (accessed November 27, 2021).

[71] European Commission. Commission Regulation (EU) No 10/2011 of 14 January 2011 on plastic materials and articles intended to come into contact with food. 10/2011 European Commission. (2011). Available at: https://eur-lex.europa.eu/legal-content/EN/TXT/?uri=CELEX:32011R0010 (accessed November 27, 2021).

[72] US Environmental Protection Agency. Chemicals Undergoing Risk Evaluation under TSCA | US EPA. Available at: https://www.epa.gov/assessing-and-managing-chemicals-under-tsca/chemicals-undergoing-risk-evaluation-under-tsca (accessed April 16, 2022).

[73] US Environmental Protection Agency. Phthalates Action Plan. Washington, DC (2012). Available at: https://www.epa.gov/sites/default/files/2015-09/documents/phthalates_actionplan_revised_2012-03-14.pdf (accessed April 16, 2022).

[74] US Food and Drug Administration. Phthalates. Available at: https://www.fda.gov/cosmetics/cosmetic-ingredients/phthalates (accessed November 27, 2021).

[75] SGS. Phthalate Regulations in the United States: An Overview. Available at: https://www.compliancegate.com/phthalate-regulations-united-states/ (accessed November 27, 2021).

[76] US Food and Drug Agency. Indirect Food Additives: Adhesives and Components of Coatings; Paper and Paperboard Components; Polymers; Adjuvants, Production Aids, and Sanitizers. Federal Register. (2022). Available at: https://www.federalregister.gov/documents/2022/05/20/2022-10531/indirect-food-additives-adhesives-and-components-of-coatings-paper-and-paperboard-components (accessed June 26, 2024).

[77] Fitzgerald E. Groups Sue to Force FDA Decision on Petitions to Ban Phthalates in Food - Earthjustice. Earth Justice. (2021). Available at: https://earthjustice.org/press/2021/groups-sue-to-force-fda-decision-on-petitions-to-ban-phthalates-in-food (accessed June 25, 2024).

[78] Maine Department of Environmental Protection. Toxics in Food Packaging Program. Available at: https://www.maine.gov/dep/safechem/packaging/index.html (accessed November 27, 2021).

[79] Bright Z. States Weigh ‘Everywhere Chemicals' Bans to Exceed Federal Rules. Bloomberg Law. (2022). Available at: https://news.bloomberglaw.com/environment-and-energy/states-weigh-everywhere-chemicals-bans-to-exceed-federal-rules (accessed July 14, 2022).

[80] Alcock RE Busby J. Risk migration and scientific advance: the case of flame-retardant compounds. Risk Anal. (2006) 26:369–81. 10.1111/j.1539-6924.2006.00739.x 16573627

[81] Government of Canada. Bill S-5, Strengthening Environmental Protection for a Healthier Canada Act - Summary of Amendments. (2023). Available at: https://www.canada.ca/en/services/environment/pollution-waste-management/strengthening-canadian-environmental-protection-act-1999/bill-c-28-strengthening-environmental-protection-healthier-canada-act-summary-amendments.html (accessed January 26, 2024).

[82] Wan Y North ML Navaranjan G Ellis AK Siegel JA Diamond ML. Indoor exposure to phthalates and polycyclic aromatic hydrocarbons (PAHs) to Canadian children: the Kingston allergy birth cohort. J Expo Sci Environ Epidemiol. (2021) 32:69–81. 10.1038/s41370-021-00310-y 33854194

[83] Government of Canada. Concentration of Contaminants and Other Chemicals in Food Composites: Total Diet Study Results. (2020). Available at: https://www.canada.ca/en/health-canada/services/food-nutrition/food-nutrition-surveillance/canadian-total-diet-study/concentration-contaminants-other-chemicals-food-composites.html#diethylhexyl (accessed April 17, 2022).

[84] Negev M Berman T Reicher S Balan S Soehl A Goulden S. Regulation of chemicals in children's products: how U.S. and EU regulation impacts small markets. Sci Total Environ. (2018) 616–617:462–71. 10.1016/j.scitotenv.2017.10.198 29127800

[85] Strategic Approach to International Chemicals Management (SAICM). Available at: https://www.saicm.org/Home/tabid/5410/language/en-US/Default.aspx (accessed December 2, 2022).

[86] United Nations Enviornment Programme. Global Framework on Chemicals. Available at: https://www.chemicalsframework.org/ (accessed October 6, 2021).

[87] European Commission. Regulation (EC) No 1907/2006 of the European Parliment and the Council of 18 December 2006 Concerning REACH. (2006). Available at: https://eur-lex.europa.eu/legal-content/EN/TXT/PDF/?uri=CELEX:32006R1907

