# Supplementary material for: Management of phthalates in Canada and beyond: can we do better to protect human health?
Source: Front Public Health. 2024 Nov 13;12:1473222. doi: 10.3389/fpubh.2024.1473222 (PMC11599199; doi:10.3389/fpubh.2024.1473222)
Supplement: Supplementary file 1 [file Table_1.docx]

**Supplementary Table 1.** Summary of key national legislation, policies, and programs controlling use of phthalates in Canada, the EU, and US

| **National legislation, policy, or program** | **Specific phthalate regulations** | **Summary of policy tool** |
| --- | --- | --- |
| **Canada** | | |
| Canadian Environment Protection Act (CEPA) | - DEHP is listed as a Schedule 1 Toxic Chemical - Completed a Screening Assessment of the Phthalate Substance Grouping with expanded cumulative risk assessment | - Legislates that HC and ECCC must maintain a register of all new and existing chemicals under Canada’s Domestic Substance List - All chemicals on the Domestic Substance List must be evaluated for toxicity - Chemicals determined to be toxic are added to the Schedule 1 Toxic Chemicals List, which allows the federal government to implement regulatory actions to minimize their risk to the environment and humans - Priority Substance Lists identify chemicals that require expedited assessments |
| Chemicals Management Plan (CMP) | - Performance Measurement Evaluations of DEHP occur every 2 years | - Formed by the HC and ECCC to review Priority Substances, including the Phthalate Substance Grouping - Operates a risk management program that monitors toxic and high priority substances |
| Cosmetics Ingredients Hotlist | - DEHP is a prohibited chemical on the Cosmetics Ingredients Hotlist | - Administrative tool employed by HC to prohibit or restrict certain substances in cosmetics |
| Canada Consumer Product Safety Act (CCPSA) | - BBP, DBP, DEHP, DiNP, DiDP, DnOP limited to <0.1% of weight in child toys | - Legislation intended to protect Canadians from healthy and safety harms posed by consumer products |
| **European Union** | | |
| Registration, Evaluation, Authorization, and Restriction of Chemicals (REACH) | - Thirteen phthalates listed on the Candidate List of Very High Concern: DEHP, DBP, BBP, DIBP, DMEP, DnHP, DHNUP, DiPP, DPP, DHEXP, DHP, 610P, DCHP - Fourteen phthalates on the Authorization List including DEHP, BBP, DBP, DiBP, DiPP, DPP, DHEXP - BBP, DBP, DEHP, DiBP limited to < 0.1% of weight in most new consumer products^[[1]](#footnote-1)^ - BBP, DBP, DEHP, DiBP, DiNP, DiDP, DNOP limited to < 0.1% of weight in child toys | - Legislates ECHA to manage toxic chemicals and protect humans and environment from exposures to harmful chemicals - ECHA must maintain a Candidate List of Substances of Very High Concern for rigorous screening - ECHA must maintain an Authorization List for chemicals that require permission for continued use after their sunset date - Annex 51 and 52 of the legislation place specific limitations on phthalates used in consumer products |
| Classification, Labelling, and Packaging (CLP) Regulation | - Class 1B toxic substances: DEHP, DBP, BBP, DiBP, DPP, DHEXP, DCHP, DiPP, DMEP - Class 2 toxic substances: DNOP | - Requires suppliers of dangerous chemicals to provide information identifying its hazards and classify the chemical appropriately - Substances may be classified as mutagenic, toxic for reproduction, or carcinogenic (Class 1A, 1B, 2) |
| Cosmetics Products Regulation | - BBzP, DBP, DEHP, DiBP are banned from use in cosmetics | - Regulates ingredients, packaging, and labelling of cosmetics |
| Food Contact Materials regulation | - Migration limits for the following food contact materials: DEHP < 1.5 mg/kg, DBP < 0.3 mg/kg , BBzP < 30 mg/kg, DiDP and DiNP combined limit of 9 mg/kg | - Authorizes use of chemicals used in food contact materials and outlines specific restrictions for their use |
| **United States** | | |
| Toxic Substances Control Act (TCSA) | - EPA’s Phthalates Action listed eight high interest phthalates requiring further assessment: DEHP, DBP, BBzP, DIBP, DiNP, DiDP, DnPP, DNOP - Seven phthalates listed on the TCSA Work Plan: DEHP, DBP, BBP, DNOP, DiDP, DiBP; none have been fully assessed yet | - TCSA enables EPA the authority to place restrictions, require reporting, enforce testing and record keeping requirements on chemical substances - EPA responsible for evaluating and monitoring safety of new and existing chemicals - TCSA Work Plan initiated to monitor existing chemicals identified as high risk |
| Food and Drug Administration (FDA) | - No restrictions placed on use of phthalates in cosmetics - Authorizes use of certain phthalates in food contact materials | - Governmental agency responsible for ensuring the safety of citizens pertaining to a wide range of products including foods, drugs, medical devices, and cosmetics |
| Consumer Product Safety Commission (CPSC) | - DEHP, DBP, BBzP, DiNP, DiBP, DPP, DHEXP, DCHP limited to <0.1% of weight in child toys | - Independent agency responsible for safety of citizens pertaining to consumer products |
| Safe Drinking Water Act | - DEHP < 0.006mg/L in drinking water | - EPA sets minimum standards for quality of drinking water |

1. Exemptions include articles for industrial or agriculture use, older motor vehicles and aircraft, lab equipment, electrical and electronic equipment, medical devices, food contact materials, articles placed on the market before July 2020 [↑](#footnote-ref-1)
